# Endobronchial Mass: A Rare Manifestation of Multiple Solitary Plasmacytoma

**DOI:** 10.5334/jbsr.3124

**Published:** 2023-05-10

**Authors:** Thanawat Anukanchanavera, Chayaporn Kaewsathorn, Nantaka Kiranantawat

**Affiliations:** 1 Prince of Songkla University, Thailand

**Keywords:** Endobronchial, plasmacytoma, extramedullary, multiple solitary plasmacytoma, endobronchial plasmacytoma

## Abstract

Multiple solitary plasmacytoma is the rare presentation of plasma cell neoplasm that can mimic multiple metastases. Primary endobronchial plasmacytoma is an extremely rare condition of extramedullary plasmacytoma. Here, we describe a case of multiple solitary plasmacytoma that initially presented with an endobronchial mass.

**Teaching Point:**

The differential diagnosis of multiple lesions in the airway is mainly metastasis and multiple solitary plasmacytoma.

## Introduction

Multiple Solitary Plasmacytoma (MSP) is a rare presentation of plasma cell neoplasm, which can mimic malignancy with multiple metastases [1]. MSP is defined as more than one localized area of clonal plasma cells of bone or soft tissue outside the bone (extramedullary) or both in the absence of bone marrow involvement or evidence of systemic disease typical for multiple myeloma [2]. Only a few cases of MSP have been documented in the literature [3,4,5,6]. Primary endobronchial plasmacytoma is an extremely rare condition of extramedullary plasmacytoma [7]. Here, we reported a case of MSP that first presented with an endobronchial mass.

## Case History

A 59-year-old male visited our hospital with a history of chronic productive cough and weight loss of 7 kg for seven months. The patient had no fever, dyspnea, or hemoptysis. He had no underlying diseases. His smoking history was about one pack-year for six years, but he had quit for two years. Laboratory results showed a slightly elevated leukocyte count of 10,700 cells/cm^3^, but the rest was unremarkable. His renal and liver function tests were within normal limits. Sputum AFB smear and culture were negative.

**Figure 1:**
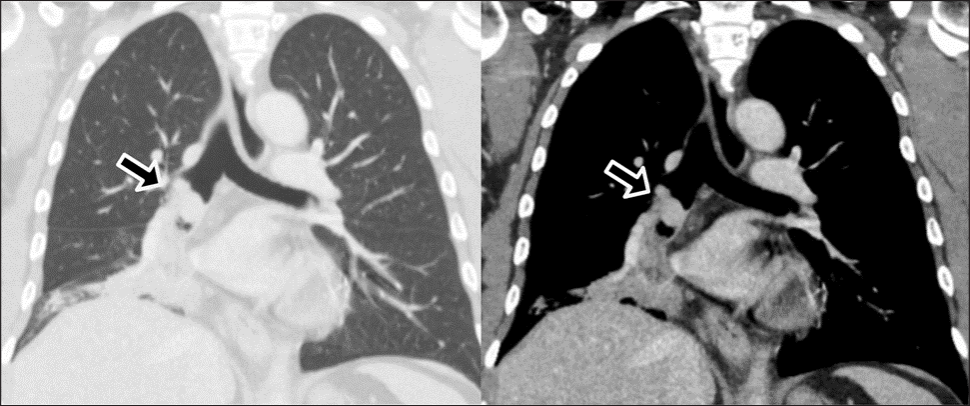
Coronal CT images demonstrating a spherical enhanced nodule (black arrow) in the bronchus intermedius causes obstructive pneumonitis in the medial segment of the right middle lobe and the basal segment of the right lower lobe.

**Figure 2:**
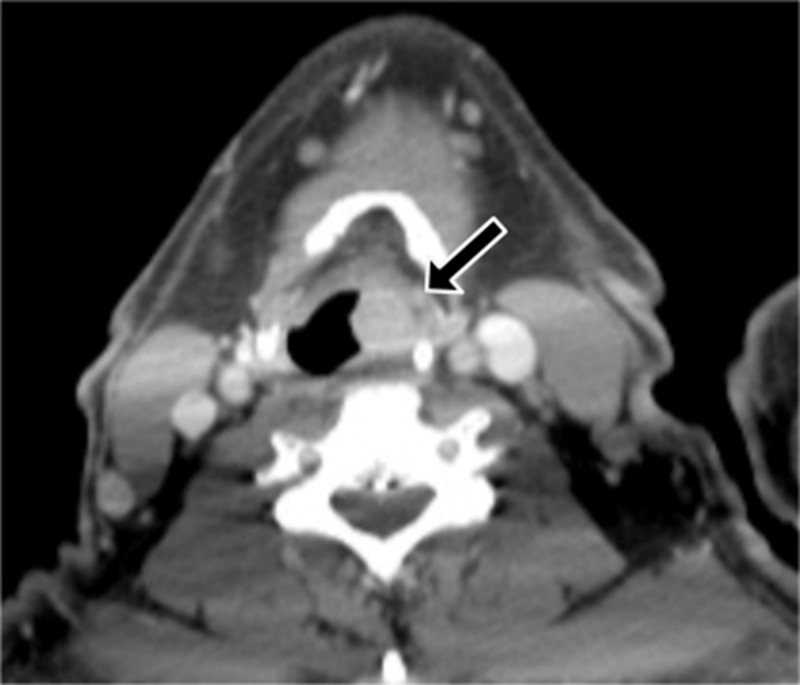
Axial CT image demonstrating a well-defined enhanced nodule (black arrow) in the left pyriform sinus.

**Figure 3:**
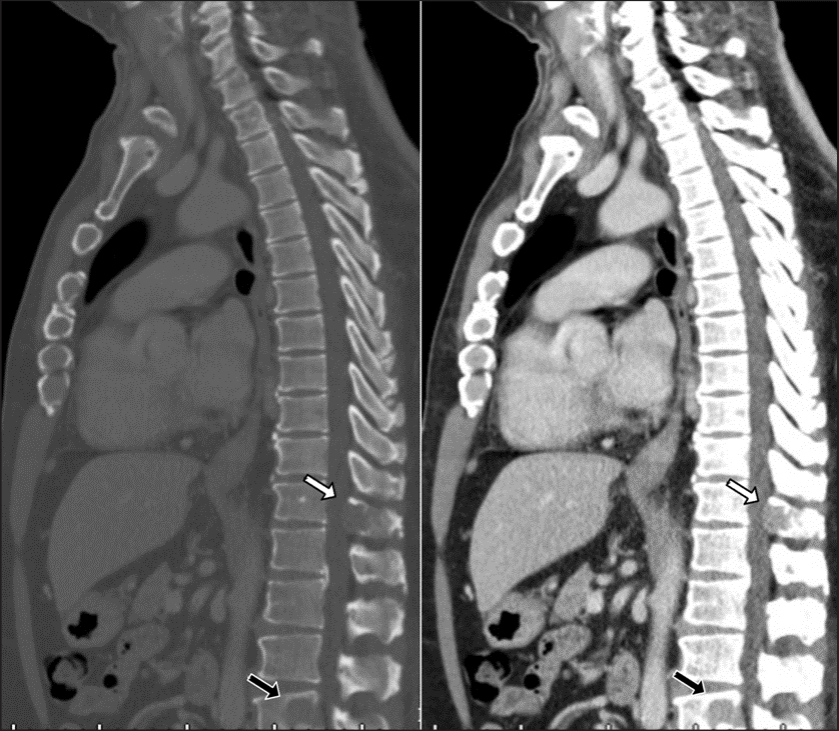
Sagittal CT images demonstrating osteolytic lesions with adjacent soft tissue component at the spinous process of T12 (white arrow) and posterior compartment of L4 (black arrow) vertebral body.

A chest radiograph revealed a focal right lower lobe consolidation. A contrast-enhanced chest computed tomography (CT) showed a round enhanced nodule obstructing the bronchus intermedius with obstructive pneumonitis in the medial segment of the right middle lobe and the basal segment of the right lower lobe (Figure 1). Another enhanced laryngeal mass at the left pyriform sinus was also found (Figure 2). Two osteolytic lesions were associated with enhanced soft tissue at T12 and L4 (Figure 3). The histopathology of the endobronchial mass in the bronchus intermedius, and the mass at the left pyriform sinus are consistent with plasmacytoma. The bone marrow biopsy revealed a normal cellular pattern without increased plasma cells.

He underwent radiation therapy at the endobronchial mass and spinal lesions, and tumor removal at the left aryepiglottic fold was also done. The patient responded well to treatment without local recurrence.

## Comments

Plasma cell neoplasms are abnormal plasma cell forming a cancerous disorder in the soft tissue of the body or bone [8]. Multiple myeloma is a plasma cell neoplasm with bone marrow and systemic involvement. In contrast, plasmacytoma is a localized proliferation of plasma cells without bone marrow involvement or evidence of systemic disease. In 2003, the International Myeloma Working Group divided plasmacytomas into solitary plasmacytoma of bone (a single bone lesion), solitary extramedullary plasmacytoma (a single soft-tissue lesion arising outside the bone), and multiple solitary plasmacytoma (multiple sites of soft tissue (extramedullary), bone or both) [2].

Extramedullary plasmacytoma (EMP) is rare, accounting for less than five percent of plasma cell neoplasms [9]. EMP manifesting as a soft tissue mass in more than one location is very uncommon. The most frequent location of EMP is the upper respiratory tract, including the nasal cavity and sinuses, nasopharynx, and larynx [2,3,4,5,6,7,8,9]. Solitary endobronchial plasmacytoma is the extremely rare manifestation of EMP, described in only a few reports [7].

In this case, the patient initially presented with nonspecific respiratory symptoms, and the chest CT showed an endobronchial mass in the bronchus intermedius that finally pathologically proven plasmacytoma. In addition, he had a mass of pathologically confirmed plasmacytoma in the left pyriform sinus, two osseous lesions, no systemic symptoms, and no bone marrow involvement. So, this patient fits the definition of multiple solitary plasmacytoma.

Endobronchial lesions could be from various causes, including tumors and tumor-like conditions. The differential diagnosis of a focal lesion that is centered within bronchi includes squamous cell carcinoma, adenoid cystic carcinoma, carcinoid, hamartoma, mucoepidermoid carcinoma, foreign body, metastasis, and others [10]. There are only metastases, and multiple solitary plasmacytoma can manifest as multiple nodules in the airways. However, radiologic investigation alone could not differentiate these indistinguishable entities, so endoscopic tissue biopsy is recommended for tissue diagnosis in which the primary lesion is unknown.

## Conclusion

Multiple solitary plasmacytoma is the rare presentation of plasma cell neoplasm, which can mimic multiple metastases. Therefore, the diagnosis of multiple solitary plasmacytoma should be differentiated when an endotracheal mass is found with a localized area of bone destruction.

## References

[1] Michels TC, Petersen KE. Multiple myeloma: Diagnosis and treatment. Am Fam Physician. 2017; 95(6): 373–84. 28318212

[2] International Myeloma Working Group (2003). Criteria for the classification of monoclonal gammopathies, multiple myeloma and related disorders: A report of the International Myeloma Working Group. Br J Haematol. 2003; 121: 749–57. DOI: 10.1046/j.1365-2141.2003.04355.x 12780789

[3] Aznab M, Khazaei M. Multifocal extramedullary and multiple solitary bone plasmacytoma: A case report and review of the literature. Int J Cancer Manag. 2019; 12(7): e91498. DOI: 10.5812/ijcm.91498

[4] Gupta S, Kumari P, Prakash S, Uttam A. Multiple solitary plasmacytoma with cutaneous manifestation: An unusual presentation – A case report. Oncol Cancer Case Rep. 2021; 7(8): 1–3.

[5] Kuchkuntla AR, Nandu NS, Husam H. Multiple solitary plasmacytoma: A rare presentation. J Oncol Res Rev Rep. 2021; 2(1): 1–3. DOI: 10.47363/JONRR/2021(2)122

[6] Dattolo P, Allinovi M, Michelassi S, Pizzarelli F. Multiple solitary plasmacytoma with multifocal bone involvement. First clinical case report in a uraemic patient. BMJ Case Rep. 2013; 2013(pii). DOI: 10.1136/bcr-2013-009157 PMC3669957 23709144

[7] Park JI, Lee YY, Lee SS, Ahn JH. A rare case of primary solitary endobronchial plasmacytoma. Thorac Cancer. 2021; 12(6): 958–61. DOI: 10.1111/1759-7714.13853 33501775 PMC7952851

[8] Pham A, Mahindra A. Solitary plasmacytoma: A review of diagnosis and management. Curr Hematol Malig Rep. 2019; 14(2): 63–9. DOI: 10.1007/s11899-019-00499-8 30788667

[9] Ooi GC, Chim JCS, Au WY, Khong PL. Radiologic manifestations of primary solitary extramedullary and multiple solitary plasmacytomas. Am J Roentgenol. 2006; 186(3): 821–7. DOI: 10.2214/AJR.04.1787 16498114

[10] Bedayat A, Yang E, Ghandili S, Pallavi Galera P, Chalian H, Ansari-Gilanif K, et al. Tracheobronchial tumors: Radiologic-pathologic correlation of tumors and mimics. Curr Probl Diagn Radiol. 2020; 49(4): 275–84. DOI: 10.1067/j.cpradiol.2019.04.003 31076268 PMC7115773

